# Artery of Percheron Infarction Presenting With Profound Autonomic Dysfunction

**DOI:** 10.7759/cureus.99830

**Published:** 2025-12-22

**Authors:** Sanihah Abdul Halim, Ahmad Zakirin Zakaria, Nur Adilah Bokti, Nur Asma Sapiai

**Affiliations:** 1 Brain and Behaviour Cluster, School of Medical Sciences, Hospital Universiti Sains Malaysia, Kubang Kerian, MYS; 2 Internal Medicine (Neurology) School of Medical Sciences, Universiti Sains Malaysia, Kubang Kerian, MYS; 3 Radiology, School of Medical Sciences, Universiti Sains Malaysia, Kubang Kerian, MYS

**Keywords:** artery of percheron, autonomic nervous system dysfunction, bilateral paramedian thalamic infarct, bradycardia, stroke

## Abstract

Artery of Percheron (AOP) occlusion is an uncommon type of stroke that results in posterior circulation infarction involving the bilateral paramedian thalami and midbrain. It can present with various clinical features. We describe a case of a male patient with multiple medical comorbidities who presented with a depressed level of consciousness accompanied by significant autonomic instability, characterized by profound bradycardia necessitating transvenous pacing. Serial brain computed tomography (CT) confirmed the presence of bilateral paramedian thalamic and midbrain infarctions within the AOP territory. This case highlights the importance of recognizing the uncommon clinical features of AOP infarction and ensuring a timely intervention in patients presenting with severe autonomic dysfunction and altered mental status.

## Introduction

The artery of Percheron (AOP) is a variant of the posterior cerebral artery (PCA) in which a unilateral PCA gives rise to paramedian perforating vessels that supply the bilateral thalami and the rostral midbrain [1]. Infarction in this territory is uncommonly reported, accounting for 0.1% to 2% of ischemic strokes [1]. Classical features include altered mental status, vertical gaze palsy, memory impairment, and fluctuating levels of consciousness. However, the presentation can be diverse and frequently misdiagnosed as metabolic or infectious etiologies. Autonomic dysfunction is an exceedingly uncommon manifestation of AOP infarction. Although the thalamus plays a pivotal role in the integration of autonomic, limbic, and cortical pathways, severe autonomic disturbances have rarely been documented as a primary clinical presentation in AOP-related strokes [2].

## Case presentation

**Figure 1:**
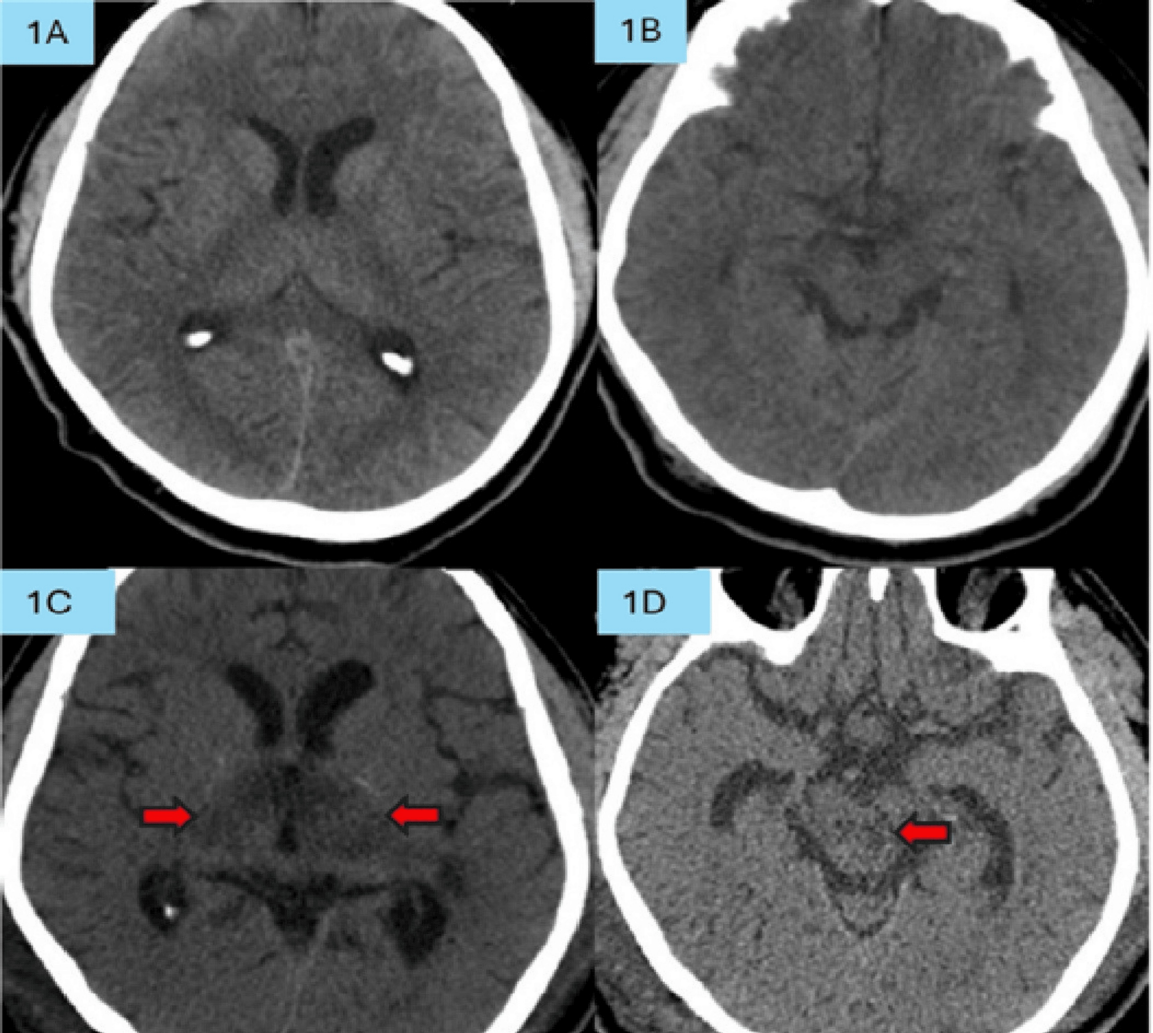
The initial brain CT scans (Figures 1A and 1B) show no evidence of infarction in the thalamus or midbrain. On repeat brain CT imaging (Figures 1C and 1D), new infarctions are noted, indicated by red arrows, involving the thalamus (Figure 1C) and the midbrain (Figure 1D).

A 62-year-old male with multiple comorbidities, including hypertension, diabetes mellitus, and chronic obstructive pulmonary disease (COPD), presented with a sudden onset of altered consciousness approximately 24 hours before admission, preceded by a four-day history of headache. No history of seizures, breathlessness, cardiac symptoms, or any sedative drug ingestion. His COPD was well controlled with medication. He was initially diagnosed with a possibility of meningoencephalitis due to the presence of fever (38°C) at the initial presentation. The emergent brain computed tomography (CT) scan (Figures 1A, 1B) was performed after symptoms had persisted for more than 24 hours, demonstrating no abnormalities in the brainstem or diencephalic region; thus, stroke was excluded.

On examination, he was drowsy but opened his eyes to call. He had minimal but relevant verbal output. He could comprehend and obey simple commands, but only after repeated instructions. He was hypertensive, with a blood pressure of 180/100 mmHg, a heart rate (HR) of 60 beats/minute (bpm), and capillary blood sugar of 9.8 mmol/L. His pupils were equal at 3 mm and reactive bilaterally. The extraocular movements were complete, and there was no gaze palsy. There was no evidence of meningeal signs. Neurological examination of the upper and lower limbs revealed normal deep tendon reflexes with down-going plantar responses. Muscle strength was 5/5 bilaterally; however, the sensory examinations could not be reliably assessed given his level of consciousness. The baseline electrocardiography (ECG) showed right bundle branch block (RBBB) without any ischemic changes.

**Figure 2:**
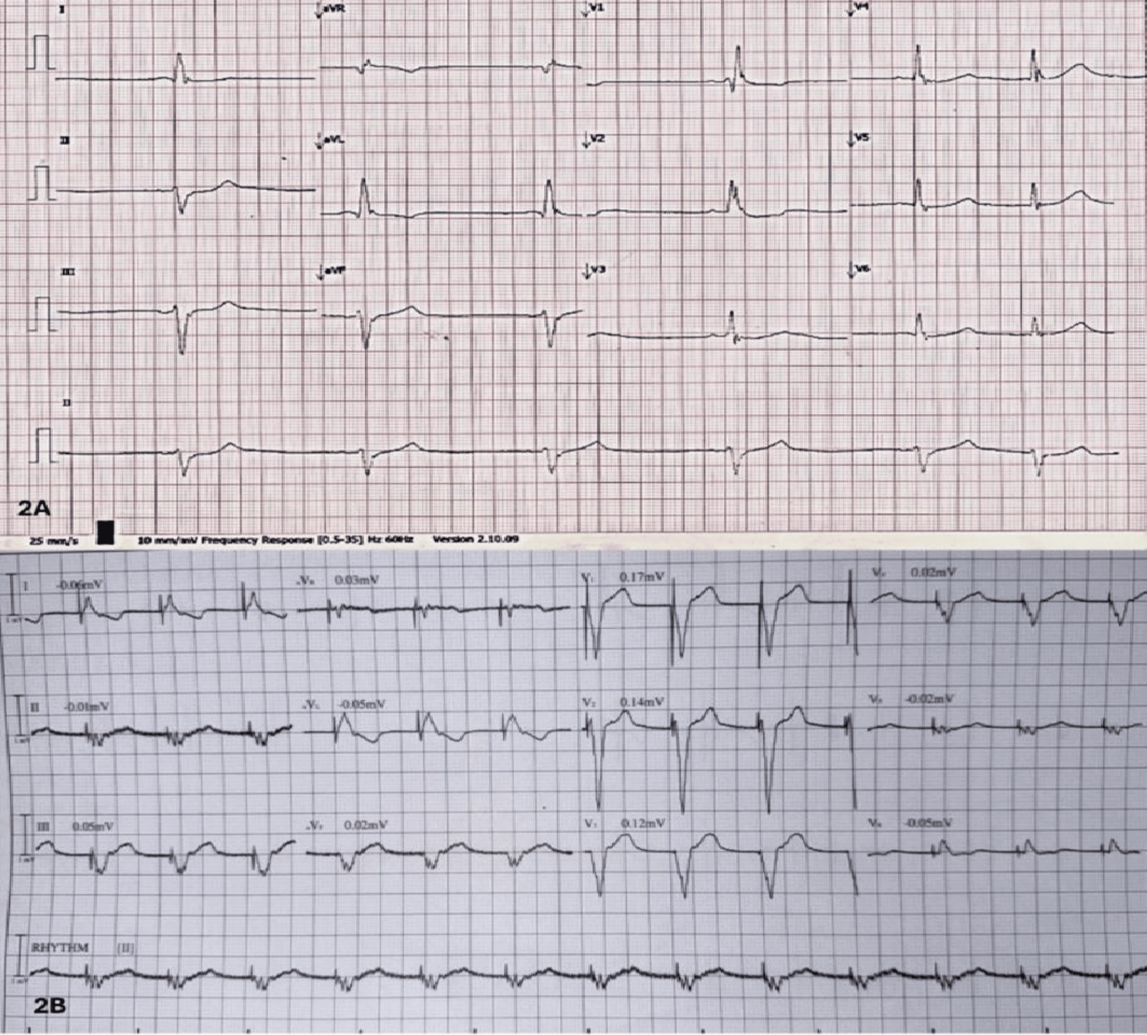
Electrocardiography shows severe bradycardia (2A) and post transvenous pacemaker insertion (2B)

While in the emergency room, the patient developed acute bronchospasm, requiring emergent endotracheal intubation. Before intubation, he became more stuporous and responded only to painful stimulation, which was attributed to hypoxic encephalopathy. Lung examination revealed crepitations in the right lower zone. Chest radiography showed findings consistent with aspiration pneumonia. He developed severe bradycardia (35-40 bpm), and a repeated ECG showed profound sinus bradycardia, HR 40 bpm (Figure 2) with persistent RBBB. Transthoracic echocardiography demonstrated normal diastolic and systolic function (ejection fraction 77%) and no evidence of regional wall motion abnormalities. Despite the administration of isoprenaline, the bradycardia persisted, necessitating the placement of a transvenous pacemaker. Baseline blood tests, electrolytes, thyroid function, C-reactive protein, blood gases, and cardiac enzymes were within normal range.

A repeat contrast-enhanced brain CT (CECT) performed over 24 hours after the stuporous episode revealed bilateral paramedian thalamic and midbrain hypodensities (Figures 1C, 1D) without contrast enhancement, consistent with an infarction in the territory of the AOP. Minor hemorrhagic transformation was present in Figure 1C but was not clinically significant. There were no radiological features of large artery occlusion or deep cerebral venous thrombosis based on contrast opacification of the vascular structures on CECT. Angiographic reconstruction was not done. Carotid ultrasound did not demonstrate any arterial stenosis. Due to hemodynamic instability, a magnetic resonance imaging (MRI) could not be performed, as the patient required high-dose inotropic support and high ventilator settings.

The patient was treated as having an autonomic dysfunction secondary to bilateral thalamic infarction. An antiplatelet agent was started as part of the standard secondary stroke prevention. Unfortunately, the patient had persistent profound hypotension and bradycardia due to autonomic instability and subsequently succumbed.

## Discussion

Occlusion of the AOP accounts for approximately 4% to 18% of all thalamic infarctions [1]. The AOP represents an uncommon anatomical variant in which a solitary paramedian thalamic-mesencephalic perforator originates from the PCA (P1) to supply both paramedian thalami and the rostral midbrain. Cardioembolic or artery-to-artery embolic events are considered the most frequent etiological mechanism in AOP-related stroke. When this vessel becomes obstructed, it typically results in bilateral paramedian thalamic infarction [3].

The thalamus functions as a principal relay center of the brain and is composed of multiple nuclear complexes, including the anterior, medial, lateral, ventral, and intralaminar nuclei [4]. These nuclei have extensive connectivity with cortical and subcortical regions. They are responsible for a wide range of neurocognitive and neurophysiological processes, including arousal, vigilance, subcortical sensory processing, memory consolidation, emotional regulation, sleep modulation, language comprehension, and multisensory integration [4].

Our patient exhibited bilateral paramedian thalamic and mesencephalic infarction. Four distinct patterns of thalamic infarction have been described on diffusion-weighted MRI: (1) bilateral paramedian thalamic infarction with mesencephalic involvement (43%), (2) bilateral paramedian thalamic infarction without mesencephalic involvement (38%), (3) bilateral paramedian thalamic infarction with concomitant anterior thalamic and mesencephalic involvement (14%), and (4) bilateral paramedian thalamic infarction with anterior thalamic involvement in the absence of mesencephalic lesions (5%) [5].

Altered consciousness in this patient reflects disruption of the paramedian thalamic nuclei and interruption of the ascending reticular activating system (ARAS) [6]. The predominant feature of autonomic dysfunction in this patient is rarely described in the context of AOP infarction [2,7]. Asavaaree et al. reported a case characterized by coma, oculomotor gaze palsy, and profound bradycardia following AOP occlusion [2]. Consistent with their observations, our patient also developed an abrupt and marked bradycardic episode in the absence of any structural cardiogenic or significant metabolic abnormalities. Bilateral thalamic infarction can disrupt descending sympathetic pathways, resulting in autonomic disturbances such as bradycardia. Several thalamic nuclei, including the zona incerta, are implicated in autonomic modulation [8].

The proposed pathophysiological mechanism involves interruption of sympathetic efferent pathways originating in the posterior thalamus, which receives afferent autonomic input from the anterior insular cortex [9]. Although the zona incerta typically derives its blood supply from independent perforators arising from the left and right posterior cerebral arteries, ischemia in this region can impair autonomic control [9]. While myocardial contractility is primarily regulated by the intrinsic sinoatrial and atrioventricular nodal conduction system, it also receives extrinsic autonomic input from higher regulatory centers, including the anterior insular cortex, posterior hypothalamus, rostral ventrolateral medulla, and the zona incerta [10].

To further substantiate our hypothesis, the impaired consciousness and bradycardia observed in our patient can be attributed to ischemic involvement of both the ascending reticular activating system (ARAS) and the zona incerta-mediated autonomic pathways within the thalamic nuclei [6,9]. The diagnosis of AOP infarction was confirmed on serial CT imaging, which initially showed no abnormalities. Still, it later demonstrated the characteristic radiographic pattern of AOP infarction.

Early diagnosis in such cases can be challenging, particularly when clinical presentation is limited to nonspecific alterations in consciousness, which may mimic septic or metabolic encephalopathy or meningoencephalitis. A key clinical indicator of ARAS-related stroke, whether at the mesencephalic or thalamic level, is the abrupt onset of neurological deterioration [6]. Additionally, this patient lacked meningeal signs, seizure activity, cognitive impairment, or focal neurological deficits that would support the diagnosis of diffuse encephalitis. The transient fever was most consistent with aspiration pneumonia, resolving promptly with antimicrobial therapy. Cardiac evaluation revealed no regional wall motion abnormalities, with preserved systolic and diastolic function and negative cardiac biomarkers, thereby excluding gross structural heart disease as the cause of severe bradycardia. Laboratory investigations, including thyroid function tests and serum electrolytes, were within normal limits, ruling out hypothyroidism and electrolyte disturbances as contributing factors.

The AOP is frequently not visualized on CT angiography (CTA) or magnetic resonance angiography (MRA) because of its diminutive caliber and deep, perforating course, thereby posing a substantial diagnostic challenge [6]. Early thalamic infarction may also be radiographically occult on initial non-contrast CT, underscoring the need for a high index of clinical suspicion [9]. As demonstrated in our case, the initial CT scan failed to reveal the infarction, highlighting the importance of avoiding premature exclusion of AOP territory stroke.

As for the etiology of AOP infarction, the patient underwent transthoracic echocardiography, which did not reveal any clot, thrombus, or cardiac embolic source. During his ICU stay, he was placed on continuous cardiac rhythm monitoring, and no tachycardia or atrial fibrillation was identified. However, these findings do not entirely exclude an embolic source, and the patient may benefit from transesophageal echocardiography and prolonged cardiac rhythm monitoring to evaluate for atrial cardiomyopathy and paroxysmal atrial fibrillation [11-13].

Although no definitive treatment exists specifically for AOP infarction beyond standard acute ischemic stroke protocols, early recognition remains crucial to facilitate timely intervention. In patients who present within the therapeutic window, intravenous thrombolysis has been associated with improved neurological outcomes. However, delayed diagnosis, often due to the non-focal, non-lateralizing clinical presentation, frequently results in missed opportunities for reperfusion therapy [11-13].

Management of AOP occlusion typically follows established secondary prevention strategies for ischemic stroke, including antiplatelet therapy, lipid-lowering agents, and a comprehensive etiologic evaluation, which is recommended for all patients with cerebrovascular events [11,13]. The prognosis of AOP infarction is variable. While many patients achieve functional recovery, others experience persistent cognitive and behavioral deficits depending on the severity of occlusion [14].

## Conclusions

This case emphasizes the importance of vigilant cardiovascular monitoring in midbrain-thalamic infarctions, where atypical autonomic features such as bradycardia may obscure diagnosis. Prompt recognition of AOP infarction is essential, as subtle or non-lateralizing presentations often delay neuroimaging and intervention. Awareness of key features such as acute consciousness impairment and autonomic instability is critical to ensure timely management and improve outcomes.
